# Photodynamic therapy mediated by methylene blue-loaded PEG accelerates skin mouse wound healing: an immune response

**DOI:** 10.1007/s10103-024-04084-1

**Published:** 2024-05-27

**Authors:** Eman Hamed, Osama Fekry Ahmed Al Balah, Mohamed Refaat, Abeer Mahmoud Badr, Ahmed Afifi

**Affiliations:** 1 Zoology Department, Faculty of Science, Cairo University, Giza, 12613 Egypt; 2 National Institute of Laser Enhanced Sciences, Cairo University, Giza, 12613 Egypt; 3 Pathology Department, Faculty of Veterinary Medicine, Cairo University, Giza, 12613 Egypt

**Keywords:** Photodynamic therapy, PEG, Methylene blue, Mice, Wound healing, Cytokines

## Abstract

**Purpose:**

Conventional approaches for enhancing wound healing may not always yield satisfactory results. Instead, we test the effectiveness of a newly developed photodynamic therapy (PDT) that uses methylene blue (MB) loaded with polyethylene glycol (PEG) (MB-PEG) hydrogel to accelerate wound healing process in mice.

**Methods:**

A dorsal skin incision with 6 mm punch which topically subjected to MB-PEG hydrogel and a low-level laser light of red light to assess the regeneration process of wounded skin. A total of 63 adult male CD1 mice divided into normal group (no treatment) and other wound groups received different treatments of laser (650 ± 5 nm and power intensity of 180 mW/cm^2^), MB-PEG, or PDT (MB-PEG followed by laser). The wound healing parameters were investigated by histological examination of the skin and measuring of proinflammatory cytokines at the early stage (48 h) and a late one on day 21. Results: at 48 h, the score of tissue granulation, inflammation, and angiogenesis process were markedly improved in wounded groups that received MB + PEG combined with laser compared to the group treated with laser alone. On day 21, a significant improvement of the inflammation was detected in the group treated with MB + PEG plus laser compared to the other groups. At 48 h, the upregulated serum levels of tumor necrosis factor (TNF)-α and interleukin (IL)-1β in the wound group were significantly (*P* < 0.001) reduced in the group treated with MB + PEG combined with laser.

**Conclusion:**

MB-PEG based hydrogel improves and accelerates wound closure in the context of laser compared to either single treatment.

**Supplementary Information:**

The online version contains supplementary material available at 10.1007/s10103-024-04084-1.

## Introduction

A skin wound results from the breakdown of the epidermal layer integrity. The wound healing process has four overlapping phases which are hemostasis, inflammation, proliferation, and remodeling [1]. Pre-clinical research on wound healing is a hot topic, especially when it comes to factors that boost healing speed or quality.

Photodynamic therapy (PDT) is one of the most brilliant therapies that used to treat malignancies, infectious disorders, and inflammatory diseases. In this type of therapy, a photosensitizer (PS) is delivered either systemically, regionally, or topically, to a patient with a lesion, followed by lighting of the lesion with visible light, which generates cytotoxic species in the presence of oxygen, resulting in cell death and tissue damage. The PDT impacts the production of inflammatory mediators such as cytokines, growth factors, and proteins [2]. The role of Low-level laser therapy (LLLT) comes to promote lymphocyte proliferation, increase fibroblast’s secretion of growth factors and enhance the uptake of both fibrin and collagen [3].

In topical PDT, the wound site should receive a sufficient concentration of PS; however, retention of liquid PS application is problematic [4]. In this approach, a fresh formulation of PS-loaded hydrogel is being researched in order to get around this issue. Many studies have attempted to address the problem of PS’s solubility; curcumin-Pluronic® F-127; [5]. Nevertheless, another research has looked into ways to improve the delivery of PS to the intended wound site; films containing chlorin p6 (Cp6, anionic PS) or methylene blue (MB), cationic PS) were prepared using sodium alginate (SA), pectin (PC), and carboxymethyl cellulose (CMC) [6]. However, a number of studies have shown that using polyethylene glycol (PEG)–protein wound dressings will enhance their hydrating action and hasten the healing process [7].

In terms of controlling the inflammatory response, PDT functions by suppressing the expression of nuclear factor-kappa B (NF-kB) and proinflammatory interleukins (IL) such as IL–1α, IL-1β, and IL-2, as well as tumor necrosis factor-α [8]. However, poly-L-lysine-conjugated chlorin p6 (pl-cp6) mediated PDT improves angiogenesis in diabetic rats [2].

Among the phenothiazine dyes, MB exhibits photosensitization with light absorption at 660 nm. It also has a benign nonallergic impact and effective against a variety of pathogens, such as viruses, fungus, and bacteria [9]. Moreover, in MB-PDT, the innate immune response against infection is mainly supported by polymorphonuclear neutrophils (PMN) that, once recruited to the infected site, it ingests and kill microbes [10], Thus, it plays an irreplaceable role in limiting infections, extending the lifespan of skin fibroblasts and enhancing cell proliferation [11].

The nanoparticles (NPs) are utilized in the delivery of non-water-soluble PSs more rapidly to the wounded part thus accelerating wound healing. The PEG is synthetic polymer that is non-toxic, inert, and appropriate for usage in medical devices [12]. The goal of this work was to create a hydrogel based on MB-PEG conjugates that could be topically given to mice with incisional skin lesions to hasten the healing process. Therefore, PEG plays a crucial role in creating a flexible synthetic hydrogel in the form of NPs. It also reduces inflammation at the onset of wound healing by modulating tissue-biomaterial interactions, which is advantageous for accelerated tissue repair and excellent cosmetic outcomes [7]. Also, the hydrogel’s efficiency was compared to each single treatment. According to our knowledge, the combination treatment of PDT with MB-PEG hydrogel might help mice’s wounds heal faster by initiating early anti-inflammatory responses.

## Materials and methods

### Hydrogel preparation (MB-loaded PEG)

According to reference [13], 40 mL of anhydrous 4-chlorophthalonitrile (Sigma-Aldrich) was mixed with 3.08 g of MB (equivalent to 5 mmol) and 2.6 g of PEG 800 (equivalent to 7.5 mmol). Subsequently, 4 g (29 mmol) of anhydrous K_2_CO3 were added. After being mixed for 24 h at a temperature of 65 °C, the reaction mixture was filtered and then diluted with dichloromethane (Sigma-Aldrich). The diluted mixture was then extracted using distilled water. In order to obtain the PEG-conjugated MB, the organic layer was dehydrated using Na_2_SO4 and concentrated. To further purify the solution, 200 mg or 1.5 mmol of zinc chloride were introduced after dissolving PEG-linked MB in a solution containing 10 mL of dimethylaminoethanol and 5 mL of n-butanol. The reaction mixture was stirred at a temperature of 100 °C for a duration of 24 h, while being exposed to nitrogen pressure. The solid returned to its original form after cooling.

### Photodynamic therapy

**Fig. 1:**
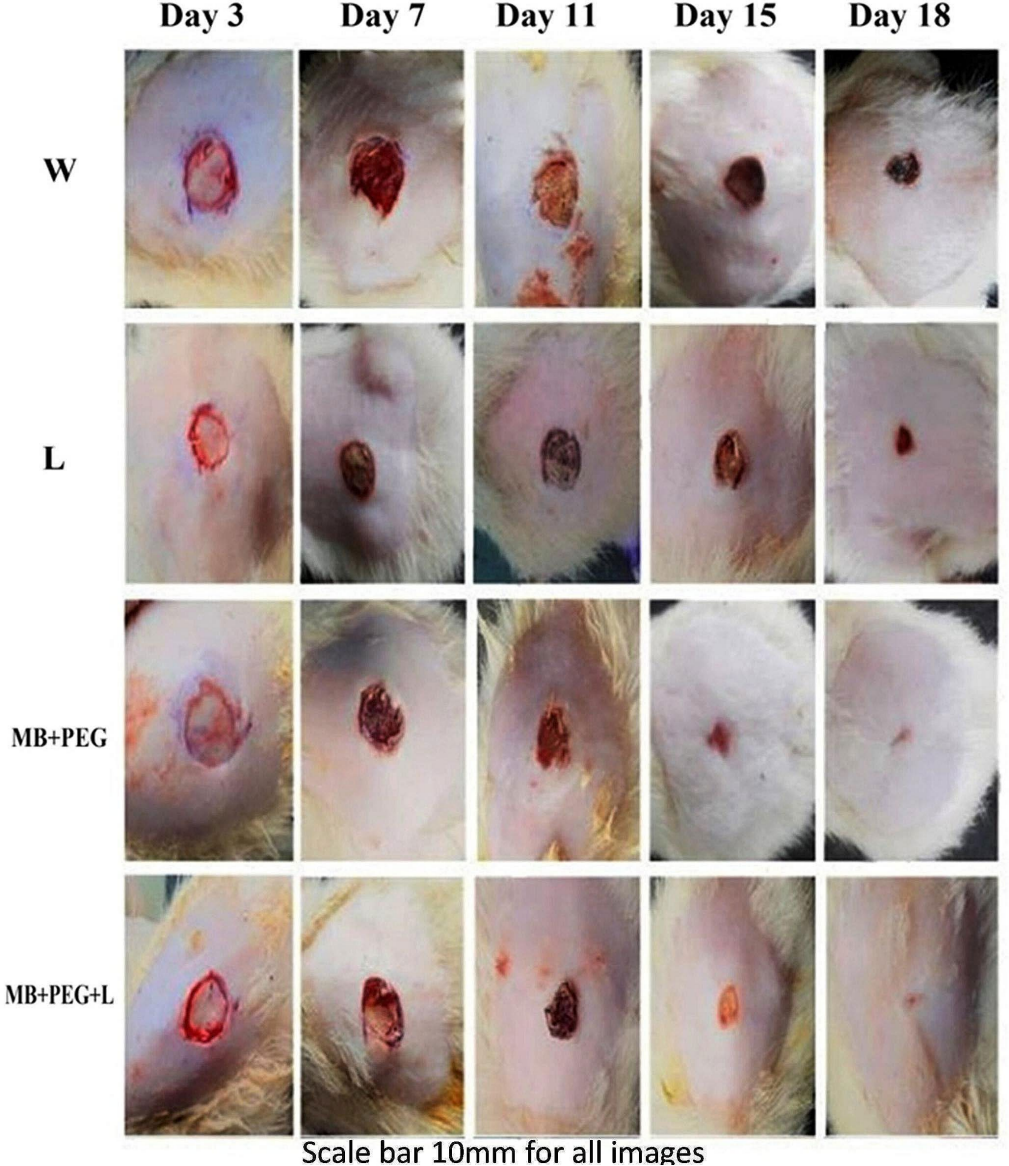
Morphological representation of mice wounds showing various phases of wound healing on days 3, 7, 11, 15, 18, and 21 post-wounding, scale bar = 10 mm

**Fig. 2:**
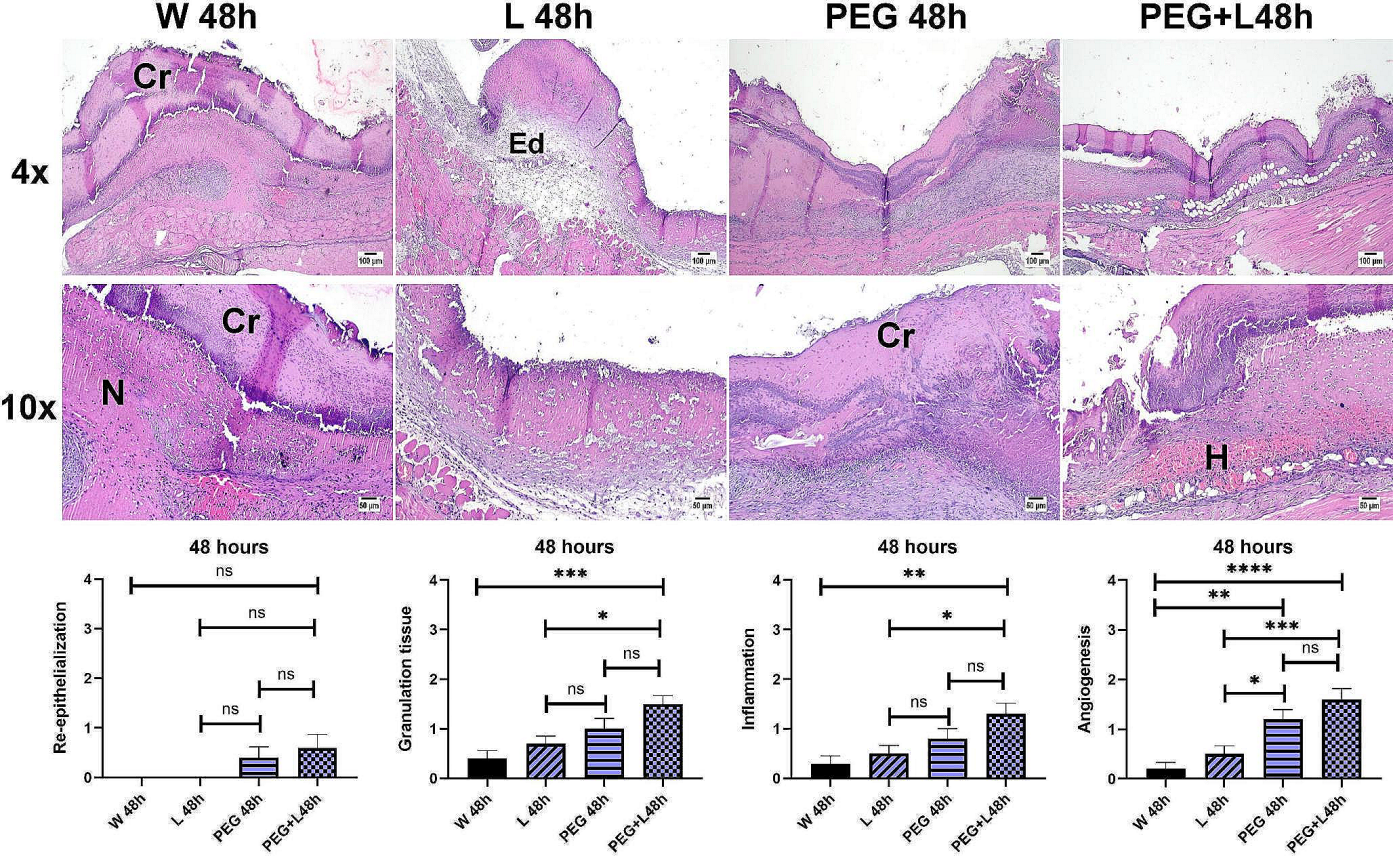
Photomicrograph of wounded areas of different mice groups at 48 h post-wounding (4X, scale bar = 100µm; 10X, scale bar = 50µm). All groups showed marked acute severe inflammation occupying the wound gap with serofibrinous exudates and necrotic crusts covering. (**Cr**) crust, (**Ed**) edema, (**H**) hemorrhages, (**N**) necrosis. Charts showing histopathological parameters of wound healing evaluation at 48 h. Data are expressed as a mean ± SEM. Significant difference was considered at *P* < 0.05. PEG group: mice treated with MB-PEG, PEG+L group: mice treated with MB-PEG+L

Mice were exposed to a diode laser (LSR-PS-ll#10,042,504 - Germany) with emitted a red laser light of wavelength 650 ± 5 nm, power intensity of 180 mW/cm^2^ (Table 1& Fig. 1S) and spot radius 6 mm at a distance of 20 cm from the injured skin part for 5 min [14]. Firstly, injured mice were treated topically with MB-loaded PEG (hydrogel) and kept in the dark for an hour to prevent MB photoexcitation and poisoning before exposure to laser (Fig. 2S).

**Table 1:** Properties of laser beam exposed to wound injury of mice

Emission Power (*P*)	Wavelength	Distance	Time	Area (A)	Intensity (I)
180 mW/mm^2^	650 ± 5 nm	20 cm	5 min	28 mm^2^	191 W/mm2

I = P/A $$A=\pi {r}^{2}$$

### Ethical consideration

A total of sixty-three male albino mice, with an average weight of 22 ± 5 g, were given normal diet pellets and had access to water *ad libitum*. They were housed in standard cages. The mice were maintained at a temperature of 22 ± 3 °C with a 12-h light/dark cycle. They were given a period of 7 days to acclimate before the experiment began [15].

### Wounding skin incision strategy

All animal groups despite the normal group were shaved at their back by an electric animal shaver to facilitate wounding [16], after that, each animal was partially numbed with isoflurane anesthetic solution (Pharco - Egypt) for a few seconds then with a punch biopsy tool (Indiamart Bb - India), a circular wound with a diameter of 6 mm was performed on each animal shaved back.

### Experimental design

A total of sixty-three mice were employed for this study, dividing them into five groups of fourteen mice each. After 48 h of wound induction, half of each group was euthanized, while the other half lived for 21 days. **Group N**: normal mice. **Group W**: injured mice receiving no treatment. **Group L**: injured mice were exposed immediately with laser light after wound induction and conducted three times a week for 21 days [17] (Fig. 2S). **Group MB-PEG**: injured mice treated immediately by topical administration of MB-PEG hydrogel after wound induction and conducted three times a week for 21 days. **Group MB-PEG + L**: injured mice treated immediately by combining a topical administration of MB-PEG hydrogel and exposure to laser after wound induction, conducted three times a week for 21 days. The diameter of the wound was measured by a caliper and recorded twice a week. The wounded area was capped during the experimental period of 21 days on days 3, 7, 11, 15, 18 and 21 as well as wound closures were compared with the original measurements (wound healing rate). The healing rate was estimated as the following calculation:

% = area before treatment - wound area after treatment / wound area before treatment.

The data are expressed as a percentage of early healing rate [18]. The animals were subjected to mild anesthesia using isoflurane [19]. Blood samples were then obtained from the venous orbital plexus of mice. Subsequently, the animals were euthanized through cervical dislocation [20].

### Histological examination

Prior to cervical dislocation, the animals were rendered unconscious through the inhalation of isoflurane [20]. The wound skin was gathered and preserved in a solution of 10% neutral buffered formalin. Washing was done in tap water then serial dilutions of alcohol (ehanol) were used for dehydration. Specimens were cleared in xylene and embedded in paraffin at 56 degrees in hot air oven for 24 h. Paraplast wax tissue blocks were prepared for sectioning at 4 microns thickness by rotatory microtome. The obtained tissue sections were collected on glass slides, deparaffinized, stained by hematoxylin & eosin stain [21], then slide sections were examined through the Olympus BX43 light microscope and photographed using the Cellsens dimensions software (Olympus) linked to Olympus DP27 camera.

### Differential blood count

Complete blood picture is performed using an automated hematology analyzer, which counts red blood cells, white blood cells (WBCs) and platelets. The concentration of hemoglobin (HG) was measured, and the red blood cell indices are calculated from the red blood cell count, average red cell volume, and HG [22].

### Cytokines assays

Quantitative determination of tumor necrosis factor (TNF)-α and interleukin (IL)-β levels [23] was performed using a sandwich enzyme-linked immunosorbent assay (ELISA) according to manufactured instructions (Glory Science Co., Ltd). Optical density was measured by a plate reader (Das, Italy) at 450 nm wavelength.

### Statistical analysis

The Statistical Package for the Social Sciences (IBM-SPSS, v.26) was used for all statistical analyses. The effect of experimental time (48 h and 21 days) on the studied parameters was applied by using a one-way analysis of variance (ANOVA). Post comparison and Duncan Multiple Range Test (DMRT) were used to detect significant differences in the intervals of the wounded groups. For all tests, data were represented as a mean ± standard error of the mean (SEM). *P* < 0.05 was considered statistically significant.

## Results

### Healing rate

The entire wound healing process was assessed on days 3, 7, 11, 15, 18 post-wounding. Images were captured to measure the wound areas and the border of each wound was traced on an image to measure the healed area (Fig. 1). The wound healing rate was significantly affected by different time points (*P* < 0.01). The wound healing rate was remarkably improved as a result of treating wounded mice with laser, MB-PEG, and MB-PEG + laser, however, group that received treatment with MB-PEG + laser showed the most effective one with a significant (*P* < 0.05/) increase in wound closure, the wound areas were entirely covered by the epidermis and the color of the wounds were close to normal skin, whereas the impact of treatment with laser alone was incomplete, and delayed till the end of experiment. Along 21days post-wounding, the healing rate (%) was significantly affected by the different time points, however, single treatment of MB-PEG or admixed with laser significantly increased the rate (%) of wound healing revealing 100% at day 21 compared to the wound group or laser group with a healing ratio of 93.28% and 98.42% respectively (Table 2).

**Table 2:** Percentage of wound healing rate in normal, wounded, and wounded treated groups at various time points

Groups	Wound healing percent					
	Day3	Day7	Day11	Day15	Day18	Day21
W	13.42^f^	35.71^d^	55.57^cd^	72.71^b^	85.00^b^	93.28^a^
L	19.85^ef^	40.42^d^	60.57^c^	77.00^b^	91.14^a^	98.42^a^
MB + PEG	24.85^e^	67.42^b^	80.71^b^	93.57^a^	99.28^a^	100^a^
MB + PEG + L	27.42^e^	56.57^c^	84.85^b^	97.28^a^	100^a^	100^a^
One-way ANOVA	*P* < 0.01					

Data are expressed as mean ± SEM based on ANOVA analysis. Means in the same row followed by the same superscript letter(s) indicates to not significantly different, while means in the same row followed by the different superscript letter(s) refers to significantly different (*P* < 0.05) according to Duncan Multiple Range Test (DMRT).

### Effects of PDT on histological architecture of skin wounds staining

Examination of 48 h post-induction of the wound was investigated in all groups. The early wound healing process was comparable in all experimental groups. The wound surface was covered by a thick crust with an underlying intense inflammatory reaction that was composed of acute inflammatory cells infiltration mainly neutrophils, edema, hemorrhage, and necrotic tissue in most circumstances. Regarding the re-epithelization score, although the application of (MB-PEG) showed early epithelial proliferation that started at the wound edges either alone or admixed with laser, no significant difference was detected between all groups. The granulation tissue and the inflammation scores showed marked a significant improvement in MB-PEG + L compared to groups W or L. The angiogenesis process showed a marked significant enhancement in groups receiving MB-PEG in comparison with other groups (Fig. 2).

**Fig. 3:**
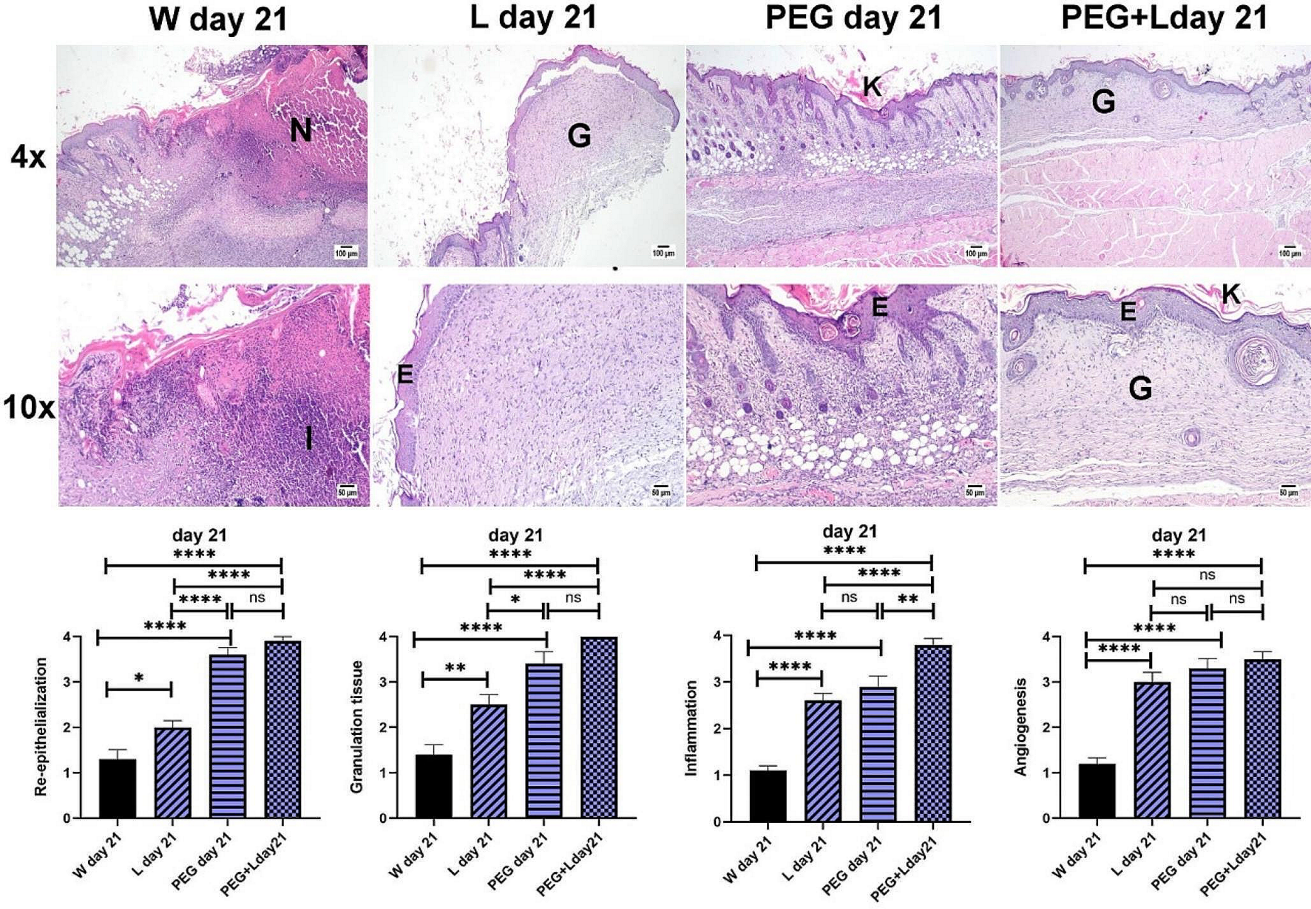
Photomicrograph of wounded areas of different mice groups after 21 days post-wounding (4x, scale bar 100 μm; 10x, scale bar 50 μm). Group W showing improper wound healing with absence of epidermal covering. Groups L, PEG and PEG + L showing compete epidermal growth and decrease the inflammation of the formed granulation tissue. Keratinization is presenting in PEG treated groups. (**E**) Epidermal layer, (**G**) granulation tissue, (**I**) inflammation, (**N**) necrosis. Charts showing parameters of wound healing evaluation at day 21. Data Expressed as means ± SEM. Significant difference was considered at *P* < 0.05. PEG group: mice treated with MB-PEG, PEG + L group: mice treated with MB-PEG + L

On day 21, group W showed poor wound healing that was characterized by incomplete epidermal remodeling with the persistence of necrotic serofibrinous crust. The wound gap was occupied by inflammatory granulation tissue which revealed excessive mononuclear inflammatory cells infiltration, necrotic debris, and aggregations of bacterial colonies. Complete wound closure was observed in group L, group MB-PEG and group MB-PEG + L which showed a newly formed epidermal covering layer and evidence of keratinization in some instances accompanied by varying grades of filling granulation tissue which showed a variable number of inflammatory cells, collagen bundles, and newly formed blood vessels. The statistical analysis showed a marked significant reduction in re-epithelization and granulation in all parameters in group L, compared to the other treated groups. Concerning re-epithelization, granulation tissue, and angiogenesis scores, the absence of significant difference was detected in the group receiving MB-PEG compared to MB-PEG + laser group. Meanwhile, a significant improvement in inflammation was detected in group MB-PEG + L compared to the other groups. The application of MB-PEG admixed with laser revealed the highest wound healing with healthy epidermal covering and organized granulation tissue (Fig. 3).

### Differential blood count

After 48 h of wound induction, the wound group revealed significantly lower values of HB% and monocytes count compared to the normal group. Treatment with laser resulted in a significant increase in the count of platelets, lymphocytes, and monocytes while group MB+PEG exhibited significantly higher values of HB% and monocytes and lower values of lymphocytes count compared to the wound group. The combination treatment of MB-PEG plus laser significantly caused an elevation in values of HB%, total count of WBC, lymphocytes, and monocytes aw well as lowered values of neutrophils compared to the wound group (Table 3).

**Table 3:** Values of blood count parameters 48 h post-wound injury in all experimental animal groups

Groups	HB%	Platelets (10^3^ /cmm)	WBCs (10^3^ /cmm)	Neutrophils	Lymphocytes	Monocytes
N	12.02$\pm$0.13^a^	706.750 ± 20.7^a^	6.4$\pm$1.07^a^	21.75 ± 5.30^a^	47.0$\pm$5.70^a^	27.25$\pm$1.10^a^
W	8.30$\pm$0.76^c^	675.2 ± 51.7^b^	6.4$\pm$1.46^a^	33.8$\pm$3.50^c^	51$\pm$4.60^b^	8.75$\pm$1.60^d^
L	8.27$\pm 0.60$^c^	1302.25 ± 281.0^c^	5.0$\pm 0.54$^a^	28.00$\pm 1.90$^b^	76.5$0\pm 1.90$^d^	10.00$\pm$2.56^b^
MB + PEG	10.82$\pm$0.65^b^	1012 ± 72.0 ^b^	5.4$\pm$0.25^a^	47.0$\pm$1.30^c^	24.5$0\pm 4.20$^a^	23.75$\pm$3.50^a^
MB + PEG + L	10.95$\pm$0.27^b^	1135.75 ± 46.0^b^	6.7$\pm$2.97^b^	17.0$\pm$2.90 ^a^	70.7$\pm 3.10$^c^	10.75$\pm 0.48$^c^

Data are expressed as a mean ± SEM based on ANOVA analysis. Means in the same row followed by the same superscript letter(s) indicates to not significantly different, while means in the same row followed by the different superscript letter(s) refers to significantly different (*P* < 0.05) according to Duncan Multiple Range Test (DMRT)

On day 21, non-significant differences were observed regarding all measured parameters in the wound group compared to the normal group. The wounded mice treated with either laser or MB-PEG showed a significant decrease in the Hb% as well as a significant increase in the count of platelets and neutrophils compared to the wound group. Combination treatment of of MB-PEG plus laser resulted in significantly higher values of platelets and neutrophils while significantly lower counts of WBCs, lymphocytes, and monocytes compared to the wound group (Table 4).

**Table 4:** Values of blood count parameters 21 days post-wound injury in all experimental animal groups

Groups	HB%	Platelets (10^3^ /cmm)	WBCs (10^3^ /cmm)	Neutrophils	Lymphocytes	Monocytes
N	12.92$\pm$0.22^a^	793.25$\pm 3.50$^a^	7.1$\pm$1.05^a^	20.25$\pm 5.5$^a^	48.00$\pm$8.70^a^	27.25$\pm$1.75^a^
W	12.02$\pm$0.13^a^	706.75$\pm 5.10$^a^	6.4$\pm$1.07^a^	21.75$\pm 5.10$^a^	47.00$\pm$5.7^a^	27.25$\pm$1.10^a^
L	11.02 ± 0.29^b^	1039$\pm 1.90$^b^	10.8$\pm 3.10$^a^	28.00$\pm 4.9$^b^	40.25$\pm 1.60$^a^	27.25$\pm$3.63^a^
MB + PEG	10.2$0\pm$0.17^d^	1284$\pm$1.20^c^	31.6$\pm$4.1^a^	35.00$\pm$9.5^d^	49.00$\pm 4.04$^a^	12.5$\pm 4.48$^c^
MB + PEG + L	11.57$\pm$1.06^c^	1171.25$\pm 2.80$^b^	5.6$\pm$0.67^b^	32.5$0\pm 3.8$^c^	39.00$\pm 2.12$^b^	23.7$\pm 5.25$^b^

Data are expressed as mean ± SEM based on ANOVA analysis. Means in the same row followed by the same superscript letter(s) indicates to not significantly different, while means in the same row followed by the different superscript letter(s) refers to significantly different (*P* < 0.05) according to Duncan Multiple Range Test (DMRT)

### Cytokines profile

A Comparison of the proinflammatory cytokines TNF-α and IL1β serum levels was investigated after 48 h and 21 days among experimental groups. At 48 h of wound injury, the serum levels of TNF-α were significantly increased (*P* < 0.001) in the wounded group compared to the normal group, while direct medication of the other groups with the action of laser, MB-PEG, or MB-PEG + laser caused a significant (*P* < 0.05, *P* < 0.01, and *P* < 0.001, respectively) decrease of TNF-α levels compared to the wounded untreated group. At the end of the study on day21, no significant differences were detected between the wounded group and the other groups with various treatments (Table 5).

**Table 5:** Serum levels of TNF-α (pg/ml) among treated groups after 48 h and 21 days

Groups	48 h	Day 21
N	431 ± 25.51	431 ± 25.51
W	733.4 ± 21.26^#^	496.9 ± 21.16
L	581.2 ± 31.84*	498.9 ± 8.963
MB + PEG	565.8 ± 36.27**	446.3 ± 14.05
MB + PEG + L	470.7 ± 45.9***	432.7 ± 12.21
*P*value	< 0.001	< 0.01
Kruskal-Wallis	18.37	11.79

Data were expressed as mean ± SEM. *n* = 6, ^#^: Significant difference (*P* < 0.001) compared to group N

*: Significant difference (*P* < 0.05) compared to the group W, **: Significant difference (*P* < 0.01) compared to group W, ***: Significant difference (*P* < 0.001) compared to group W

During the first 48 h, the IL-1β serum levels in the wounded group showed significantly higher levels (*P* < 0.001) than those of the normal group while the wounded groups treated with laser, MB-PEG, or MB- PEG + laser exhibited significantly lower levels (*P* < 0.05, *P* < 0.01, and *P* < 0.001) respectively than those wounded group. On the other hand, at the end of the study, IL-1β still showed significantly higher levels (*P* < 0.001) in the wounded group compared to the normal group but all other groups that received various treatments showed no significant differences compared to the wounded group (Table 6).

**Table 6:** Serum levels of IL-1β (pg/ml) in normal, wounded, wounded treated groups after 48 h and 21 days

Groups	48 h	Day 21
**N**	70.96 ± 4.93	70.96 ± 4.93
W	198.8 ± 37.76^#^	62.22 ± 3.267^#^
L	142.8 ± 20.93*	65.77 ± 4.9
MB + PEG	59.66 ± 3.744**	59.91 ± 6.269
MB + PEG + L	50.84 ± 4.227***	52.34 ± 4.156
*P*value	*P* < 0.001	*P* ≥ 0.05
Kruskal-Wallis	40.32	19.1

Data were expressed as a mean of 6 mice ± SEM. #: Significant difference (*P* < 0.001) compared to group N, *: Significant difference (*P* < 0.05) compared to the group W, **: Significant difference (*P* < 0.01) compared to group W, ***: Significant difference (*P* < 0.001) compared to group W

## Discussion

Healing process following tissue injury includes homeostasis, inflammation, proliferation, and tissue remodeling phases [24]. Achieving the fastest healing rate and reepithelization is the aim of this investigation. In topical PDT, a crucial stage in treating wound site is delivering PS at the right concentration. As a result, PS must be applied in the right formulation at the affected site [6]. The recently developed MB-PEG hydrogel outperformed all expectations in terms of its ability to promote wound healing and reepithelialization. The role of MB comes to reduce inflammation and protect against free radicals, thus, it hastens the healing process [25], while the PEG-based hydrogel showed good biocompatibility and safety [26]. After applying the MB-PEG hydrogels, bleeding from the incisions on the dorsum of mice immediately stopped, and the wound openings closed within few min while the wound openings did not close after applying laser only. Along 21 day, both groups that treated with MB-PEG hydrogel and MB-PEG + laser recorded highest percent of wound healing rate and the wounds have a distinct appearance from a macroscopical and histological perspective. The impact of PEG-based chitosan (hg-PEGDA-Q) was also thoroughly examined by [27] who suggested that hydrogel scaffolding networks could be a therapeutic substitute to quicken the healing process in mice under both normal and diabetic settings. Also, according to [28], the polyethylene glycol/triethoxysilane-modified polyurethane (PUESi) dressing improved wound healing in rats by generating micronegative pressure through its high absorption capacity with deformation, in addition to being made using an easy-to-use and effective technique.

Histologically, 48 h post-wounding; the inflammation score of infiltrated neutrophils, edema, hemorrhages and necrotic tissue was significantly elevated in the groups treated with laser, MB-PEG hydrogel or MB-PEG + laser compared to wounded group, During the inflammation phase, the granulation tissue is primarily composed of predominant inflammatory cells, mainly neutrophils that are recruited to the wound site and removed during the repair process. However, during the proliferative phase, endothelial cells, macrophages, and fibroblasts begin to fill the wound area to restore tissue integrity [24]. Twenty-one days post-wounding, the best rate of wound healing with a healthy epidermal layer and well-organized granulation tissue was demonstrated by the group treated with MB + PEG hydrogel or admixed with laser. Several studies approved that PDT improves angiogenesis and tissue healing, including regular epithelial lining, decreased fibrinous exudate, and more organized and thick conjunctive tissue, which resulted in full re-epithelization and keratin production [2, 29–31]. The outcomes of this investigation were corroborated with [32] who concluded that topical application of low dose Foslip® in a collagen matrix followed by illumination considerably accelerates wound healing.

Among the hematological parameters, the HG% was significantly decreased either in laser treated group or MB-PEG + laser group compared the wounded and normal group at late phase of recovery (day21). A previous study denoted to the decrease in the erythrocytes volume by the exposure to LLLT [33]. This is because LLLT can split cell membranes, increasing porosity and attracting calcium ions that are free in extracellular solution and move to the intracellular fluid of the erythrocytes. Thus, this rise causes the passage of K + ions into the extracellular fluid, which causes a decrease in the MCV of erythrocytes [34]. White blood cells are key players in inflammation because they operate as phagocytes in the tissue, removing bacteria and cellular debris. After 48 h of injury, the total count of WBCs of wounded mice treated with both laser and MB-PEG hydrogel significantly promoted higher counts of WBCs while at the end of study by day 21 counteract the increase of the count compared to wounded or normal group. These findings may denote to the effect of photodynamic therapy in stimulation of WBCs production in early phases while after a long period of photodynamic therapy has inverse effect by decreasing the WBCs count. In a previous study, an increase in leukocytes was linked to the elevation in mitochondrial intracellular ATP [35]. Neutrophils play a crucial role in the early stages of inflammation by helping to restore hemostasis, perform phagocytosis, and release extracellular chemical messengers. They are attracted to wound sites in huge numbers, where they release cytokines and toxic substances that foster an inflammatory environment. Inflammation reaches its peak 48 h after injury, and during this period, the site of injury begins to malfunction and become red, hot, swollen, and painful. Differential count of neutrophils showed significant higher levels in wounded mice compared to the normal group after 48 h of injury. These findings are comparable with [36] and [37] who showed that in full-thickness incision wounds in mice displayed infiltration of the tissue by neutrophils and macrophages for at least 13 days. Whereas application of MB-PEG or MB-PEG + laser along 21 days significantly induced a rise in the count of neutrophils compared to wounded and normal groups, these results are compatible with [10] who found that in MB mediated PDT; human neutrophils adhesion increases and does not modify myeloperoxidase release. Furthermore, application of MB-PEG+laser promoted a significant reduction in the count of lymphocytes compared to wounded group. These findings are consistent with [38], who discovered that the mitogen phytohemagglutinin significantly suppressed lymphocyte proliferation after incubating lymphocytes for seven days.

In the context of PEG-functionalized NPs, polymeric forms with dimensions of less than 31.5 nm could effectively avoid immune cells, while those larger than 50.2 nm triggered anti-PEG [39]. In this study, the dimension of PEG NPS used ranged from 25 to 30 nm which in turn facilitates penetration through the membrane and improves the distribution of the ROS, hence improving the efficacy of anti-inflammation PDT.

Regarding to the levels of the proinflammatory cytokines, the study’s findings confirmed the hypothesis by showing that 48 h-post wounding, whereas MB-PEG hydrogel when topically applied to incisional wounds individually or admixed with laser, it can diminish the levels of TNF-α and IL-1β in vivo. This sequestration action causes a markedly decreased input of immune cells influx into the wound [40]. The decreased inflammatory signaling pathway enhances the wound environment, which in turn stimulates vascularization, re-epithelialization, and granulation tissue development. The approach using PEG in inflammatory lung injury in humans has been reported by [41]. They found that PEG has the ability to combine many endothelial cell barrier-regulatory chemicals to quickly activate signal transduction pathways that enhance barrier function and target the cytoskeleton. In addition, MB has long been known for its anti-inflammatory qualities [9]. The capacity of MB in decreasing both STAT3 activation and the serum levels of IL-6 in LPS-administered mice have suggested important mechanisms underlying the effects of MB on inflammation [42].

Low level laser therapy (LLLT) has been shown to be successful in treating inflammation in a number of investigations, and these studies also show that laser light has the ability to modulate pro- and anti-inflammatory mediators [43&^46^]. Groups that were administered with LLLT 48 h after injury showed a statistically significant decrease in the expression of IL-1β and TNF-α levels protein when compared with the non-treated wound group, but 21 days after injury the statistical analysis returned to levels similar to that of the normal group [47]. It has been suggested that LLLT with 50 mW was efficient in modulating inflammatory mediators (IL-1β and IL-6) and inflammatory cells (macrophages and neutrophils), which correlated with the histology that showed a reduction in the inflammatory process. However, in this investigation, the outcomes obtained from treating with MB-PEG hydrogel were superior to those obtained from treating with LLLT, both histologically and physiologically, as a result, the inflammatory phase and the remodeling phase begin earlier in MB-PEG based hydrogels treatment.

## Conclusion

Based on the data collected, it appears that MB-PEG hydrogel facilitates the healing process of acute wound damage. When LLLT is applied in combination with MB-PEG hydrogel, the healing rate is increased and the skin’s morphological and histological characteristics are improved. However, the effects of LLLT alone on inflammation and wound closure are somewhat less favorable.

## Electronic supplementary material

Below is the link to the electronic supplementary material.

Supplementary Material 1: A photograph of lab adjustable laser power supply — diode laser (LSR-PS-ll#10,042,504)

Supplementary Material 2: A photograph of the mouse with injured skin during exposure to laser diode
