# Correction to: Photodynamic therapy mediated by methylene blue-loaded PEG accelerates skin mouse wound healing: an immune response

**DOI:** 10.1007/s10103-024-04160-6

**Published:** 2024-08-01

**Authors:** Eman Hamed, Osama Fekry Ahmed Al Balah, Mohamed Refaat, Abeer Mahmoud Badr, Ahmed Afifi

**Affiliations:** 1 Zoology Department, Faculty of Science, Cairo University, Giza, 12613 Egypt; 2 National Institute of Laser Enhanced Sciences, Cairo University, Giza, 12613 Egypt; 3 Pathology Department, Faculty of Veterinary Medicine, Cairo University, Giza, 12613 Egypt

**Correction to: Lasers Med Sci**

10.1007/s10103-024-04084-1

In the published paper, the authors regret to inform that a mistake was made in the author’s name Osama Fekry as it was written in mistake as Osama Fakry.

The original article has been corrected.

