# Supplementary figures and images for: Photodynamic therapy mediated by methylene blue-loaded PEG accelerates skin mouse wound healing: an immune response
Source: Lasers Med Sci. 2024 May 27;39(1):141. doi: 10.1007/s10103-024-04084-1 (PMC11129982; doi:10.1007/s10103-024-04084-1)

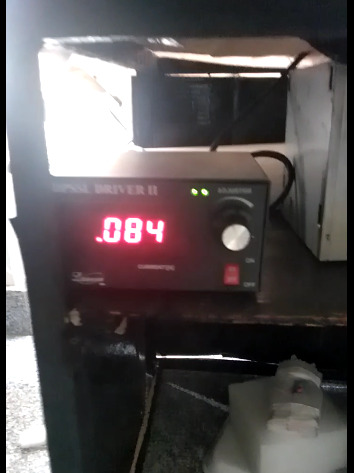

Supplement: Supplementary file 1 — Supplementary Material 1: A photograph of lab adjustable laser power supply — diode laser (LSR-PS-ll#10,042,504) [file 10103_2024_4084_MOESM1_ESM.jpg]

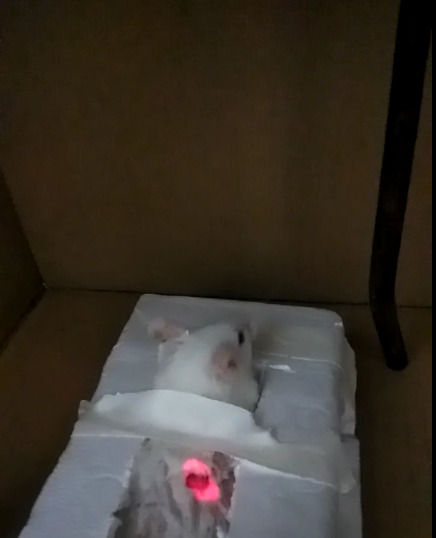

Supplement: Supplementary file 2 — Supplementary Material 2: A photograph of the mouse with injured skin during exposure to laser diode [file 10103_2024_4084_MOESM2_ESM.jpg]
